# All-Cause and Cause-Specific Mortality Among Patients With Narcolepsy

**DOI:** 10.1001/jamanetworkopen.2025.36771

**Published:** 2025-10-09

**Authors:** Chih-Wei Hsu, Yen-Shan Yang, Yang-Chieh Brian Chen, Liang-Jen Wang, Mu-Hong Chen, Yao-Hsu Yang, Chih-Sung Liang, Edward Chia-Cheng Lai

**Affiliations:** 1 Department of Psychiatry, Kaohsiung Chang Gung Memorial Hospital, Chang Gung University College of Medicine, Kaohsiung, Taiwan; 2 Department of Psychiatry and Behavioral Sciences, The University of Texas Health Science Center at Houston, Houston; 3 Department of Child and Adolescent Psychiatry, Kaohsiung Chang Gung Memorial Hospital, Chang Gung University College of Medicine, Kaohsiung, Taiwan; 4 Department of Psychiatry, Taipei Veterans General Hospital, Taipei, Taiwan; 5 Department of Psychiatry, College of Medicine, National Yang Ming Chiao Tung University, Taipei, Taiwan; 6 Health Information and Epidemiology Laboratory, Chang Gung Memorial Hospital, Chiayi County, Taiwan; 7 Department of Psychiatry, Beitou Branch, Tri-Service General Hospital, National Defense Medical University, Taipei, Taiwan; 8 Department of Psychiatry, National Defense Medical University, Taipei, Taiwan; 9 School of Pharmacy, Institute of Clinical Pharmacy and Pharmaceutical Sciences, College of Medicine, National Cheng Kung University, Tainan, Taiwan; 10 Population Health Data Center, National Cheng Kung University, Tainan, Taiwan

## Abstract

**Importance:**

Narcolepsy is a sleep disorder potentially affecting mortality, yet evidence on this association remains sparse.

**Objective:**

To examine whether narcolepsy is associated with an increased risk of all-cause and cause-specific mortality.

**Design, Setting, and Participants:**

This retrospective cohort study used data from the Taiwan National Health Insurance Research Database (NHIRD) from 2001 to 2021, with patients followed up until death or December 31, 2022. Patients were aged 6 years or older with 2 or more narcolepsy diagnoses from psychiatrists or neurologists. Controls were selected from the NHIRD as a population-based sample. Controls without narcolepsy were matched in a 1:4 ratio on sex and birth date (±6 months). Sibling controls were siblings without narcolepsy. Statistical analysis was performed from January to April 2025.

**Exposures:**

Clinical narcolepsy diagnosis, confirmed via NHIRD records (*International Classification of Diseases, Ninth Revision, Clinical Modification* code 347 or *International Statistical Classification of Diseases and Related Health Problems, Tenth Revision, Clinical Modification* code G47.4).

**Main Outcomes and Measures:**

The primary outcome was all-cause mortality, measured as hazard ratios (HRs) using Cox proportional hazards regression, adjusted for birth year, sex, income, urbanization, and Charlson Comorbidity Index. Secondary outcomes included cause-specific mortality (natural, unnatural, accidents, suicides).

**Results:**

Of 3187 patients with narcolepsy (mean [SD] age, 29.5 [16.1] years; 1674 male patients [52.5%]) and 12 748 controls (mean [SD] age, 29.5 [16.1] years; 6696 male patients [52.5%]), 132 patients with narcolepsy and 456 controls died. Psychiatric comorbidities, especially depression (1167 of 3187 [36.6%] vs 861 of 12 748 [6.8%]) and anxiety (1054 of 3187 [33.1%] vs 853 of 12 748 [6.7%]), were more common in the narcolepsy group than in the control group. Crude all-cause mortality rates were 44.3 per 10 000 person-years among patients with narcolepsy and 38.1 per 10 000 person-years among controls. All-cause mortality was not increased among patients with narcolepsy (HR, 0.96; 95% CI, 0.79-1.17). There was no increase among patients with narcolepsy in cause-specific mortality for natural causes (HR, 0.90; 95% CI, 0.73-1.11), unnatural causes, (HR, 1.41; 95% CI, 0.83-2.40), accidents (HR, 1.37; 95% CI, 0.64-2.95), and suicides (HR, 1.41; 95% CI, 0.62-3.22). The sibling cohort analysis similarly demonstrated no significantly increased risk among patients with narcolepsy of all-cause mortality (HR, 1.14; 95% CI, 0.63-2.06) or cause-specific mortality from natural causes (HR, 0.66; 95% CI, 0.28-1.56), unnatural causes (HR, 2.08; 95% CI, 0.87-4.98), accidents (HR, 1.61; 95% CI, 0.48-5.37), or suicides (HR, 3.43; 95% CI, 0.88-13.28).

**Conclusions and Relevance:**

In this cohort study of Taiwanese residents, narcolepsy was not associated with excess all-cause or cause-specific mortality. These findings reduce immediate clinical concern, but the wide 95% CIs suggest that a modest increase in risk cannot be excluded; replication in other populations with longer follow-up is warranted.

## Key Points

**Question:**

Is narcolepsy associated with an increased risk of all-cause and cause-specific mortality?

**Findings:**

In this cohort study of 3187 patients with narcolepsy, neither all-cause nor cause-specific (natural, unnatural, accidental, or suicide) mortality was elevated compared with 12 748 sex- and age-matched controls or 3287 siblings without narcolepsy.

**Meaning:**

These findings suggest there is no association between narcolepsy and mortality; however, the findings warrant replication in diverse settings with continued clinical surveillance.

## Teaser

This cohort study uses data from the Taiwan National Health Insurance Research Database to assess whether narcolepsy is associated with an increased risk of all-cause and cause-specific mortality.

## Introduction

Narcolepsy is a lifelong neurologic disorder characterized primarily by excessive daytime sleepiness and frequently accompanied by cataplexy, an abrupt reduction in muscle tone triggered by emotional reactions.^1^ Additional symptoms include disrupted nocturnal sleep, hallucinations at sleep onset or awakening, and transient paralysis episodes.^2^ Typically manifesting in adolescence or early adulthood,^3^ narcolepsy significantly affects daily functioning, quality of life, and health care use,^4,5^ necessitating ongoing medical care. The global prevalence of narcolepsy is approximately 30 per 100 000 individuals, with higher rates in regions such as Japan and lower rates elsewhere, particularly in other parts of Asia.^6,7^

The question of whether narcolepsy is associated with increased mortality remains largely unresolved, as only a limited number of studies have specifically explored mortality outcomes among these patients, yielding inconsistent results. Some research suggests a potentially increased risk of death among patients with narcolepsy,^8^ while others have found no significant differences compared with general populations.^9^ These prior investigations often face methodological limitations, such as small sample sizes^9^ or the absence of well-defined control populations.^8^ Previous literature has rarely analyzed specific mortality causes among individuals with narcolepsy, leaving uncertainty about whether any elevated mortality risk might be associated with natural causes or unnatural causes, such as accidents and suicides. Prior studies have documented significantly elevated risks for traffic accidents among individuals with narcolepsy compared with the general population,^10^ along with an increased prevalence of psychiatric comorbidities, particularly depression and suicidal ideation, which affect approximately 15% to 20% of patients with narcolepsy.^11,12^ Despite recognizing these potential risk factors, previous studies have yet to conclusively determine whether these observed risks are definitively associated with higher mortality rates.

This study aimed to evaluate all-cause and cause-specific mortality risks associated with narcolepsy by comparing patients with matched controls without narcolepsy from the general population as well as with siblings without narcolepsy. The use of sibling cohorts provides a methodological advantage by implicitly controlling for genetic and early environmental factors. Using nationwide registry data with extensive follow-up enables the robust evaluation of long-term mortality outcomes, systematically adjusting for demographics and medical comorbidities. By addressing limitations of previous research, this rigorous, population-based approach clarifies whether narcolepsy is independently associated with mortality, distinguishing clearly between natural and unnatural causes of death, including accidents and suicides.

## Methods

### Data Sources

This retrospective cohort study was approved by the institutional review board of Chang Gung Memorial Hospital in Kaohsiung, Taiwan, which waived the need for informed consent because the underlying data had been fully anonymized before release. The study was conducted in accordance with the Declaration of Helsinki^13^ and the Strengthening the Reporting of Observational Studies in Epidemiology (STROBE) reporting guideline.^14^

Data were obtained from the Taiwan National Health Insurance Research Database (NHIRD), a nationwide claims database covering more than 99% of Taiwan’s population since 1996.^15^ The NHIRD includes comprehensive demographic details (eg, sex, date of birth, insurance premium based on personal income level, residential area, and the familial relationships between policyholders and their dependents), records of outpatient and inpatient medical visits (dates of visits and associated diagnoses), and records from the national death registry (date and cause of death). All personal identifiers are encrypted, allowing for the secure linking of records across datasets without revealing identities. The data used spanned from 2000 through 2022. Health conditions were identified using codes from the *International Classification of Diseases, Ninth Revision, Clinical Modification* (*ICD-9-CM*) (2000-2015) and the *International Statistical Classification of Diseases, Tenth Revision, Clinical Modification* (*ICD-10-CM*) (2016-2022). The eFigure in Supplement 1 shows the research flowchart. Previous observational studies have demonstrated the robustness of the NHIRD for epidemiologic research.^15,16^

### Matched and Sibling Cohorts

Patients with narcolepsy were defined as individuals who had received at least 2 clinical diagnoses of narcolepsy (*ICD-9-CM* code 347 or *ICD-10-CM* code G47.4) from a psychiatrist or neurologist within the study inclusion period, spanning January 1, 2001, through December 31, 2021.^17^ In addition, we restricted the narcolepsy cohort to individuals aged 6 years or older at the time of initial diagnosis to further reduce the risk of misdiagnosis among very young children. A matched comparison cohort consisting of individuals without narcolepsy was subsequently constructed. For each patient with narcolepsy, 4 matched control individuals without any record of narcolepsy diagnoses within the entire NHIRD database period were selected from the general population. Controls were matched individually with patients with narcolepsy based on identical sex and similar birth dates (within a 6-month range). Simultaneously, we created a sibling cohort designed to enable within-family comparisons. In this cohort, we first identified all patients with narcolepsy who had at least 1 sibling enrolled in the NHIRD. Siblings were defined explicitly as individuals sharing at least 1 biological parent, as indicated in the insurance database. Within these identified families, the sibling cohort included the index patients with narcolepsy as well as their biological siblings without narcolepsy. Participants from both the matched control cohort and the sibling cohort were followed up from their respective index dates, defined as the date of the first confirmed narcolepsy diagnosis for matched cohort participants or the beginning of available NHIRD data coverage for sibling cohort participants. Follow-up continued until either the date of death or the conclusion of the study period on December 31, 2022, whichever occurred first.

### Study Outcomes and Covariates

For study participants whose deaths occurred between 2001 and 2022, we obtained data regarding all-cause mortality as the primary outcome, along with detailed information on specific underlying causes of death as secondary outcomes from the NHIRD Cause of Death Registry.^18^ The underlying causes of death were categorized as either natural or unnatural, with accidental and suicide deaths identified within the unnatural category. To ensure confidentiality and minimize potential identification of individuals, data regarding deaths were extracted only if the total number exceeded 3 within any given category.

For each participant, we collected baseline demographic information, including age at cohort entry, sex, income level, degree of urbanization at their residential location, and psychiatric and medical comorbidities. Individual income status was stratified into quartiles—highest (>25%), upper-middle (25%-49%), lower-middle (50%-74%), and lowest quartile (≤75%)—with reference to the matched controls. The income data of matched controls provided a representative benchmark for the general income distribution of the population within the database. Residential urbanization was classified into 4 hierarchical levels (1-4, from most to least urbanized), serving as an indirect measure of health care accessibility in Taiwan.^19^ These urbanization categories were established based on factors including local population density, demographic age distribution, proportion of agricultural employment, prevalence of postsecondary education, and availability of medical professionals.^19^ Psychiatric comorbidities were systematically classified into 9 diagnostic groups consisting of neurodevelopmental disorders (including intellectual disability, autism spectrum disorders, attention-deficit/hyperactivity disorder, Tourette syndrome, and chronic tic disorder), psychotic disorders, bipolar disorders, depressive disorders, anxiety disorders, obsessive-compulsive disorders, eating disorders, substance use disorders, and personality disorders.^20^ Comprehensive lists of corresponding *ICD-9-CM* and *ICD-10-CM* diagnostic codes are presented in eTable 1 in Supplement 1. Medical comorbidities were quantified using the Charlson Comorbidity Index (CCI), a validated index representing overall physical health burden.^21^ The CCI incorporates a total of 22 medical conditions, namely, dementia, cerebrovascular disease, hemiplegia, paraplegia, chronic pulmonary disease, myocardial infarction, congestive heart failure, peripheral vascular disease, peptic ulcer disease, liver disease (classified as mild, moderate, or severe), diabetes (controlled and uncontrolled), kidney disease, rheumatologic disease, malignant neoplasms (localized and metastatic), leukemia, lymphoma, and AIDS.^21^

### Statistical Analysis

Statistical analysis was performed from January to April 2025. Baseline characteristics were summarized for each cohort; continuous variables were expressed as mean (SD) values, and categorical variables were expressed as counts and percentages. Kaplan-Meier curves depicted survival over time, and the cumulative incidences with risk differences for all-cause mortality were calculated at yearly intervals. Cox proportional hazards regression models were used to compute hazard ratios (HRs) and corresponding 95% CIs for mortality risk, comparing patients with narcolepsy and matched controls, with time since cohort entry as the underlying time scale. These analyses were initially conducted using all-cause mortality as the outcome and subsequently repeated separately for each specific cause of death. The first model (model 1) provided a crude, unadjusted HR. In the second model (model 2), we adjusted for birth year, sex, income level, urbanization level, and CCI. Missing data for these covariates were categorized as unknown and included in the models as nominal variables.

We performed 3 sensitivity analyses. First, to address potential familial confounding, we compared patients with narcolepsy with their siblings without narcolepsy. Second, considering that the COVID-19 pandemic could have affected mortality risk among participants, we redefined the study end point date as December 31, 2019. Third, we conducted a complete-case analysis by excluding participants who had missing data for selected covariates. For all the sensitivity analyses, we repeated the model 1 and model 2 analyses. Furthermore, in the sibling cohort analysis, sibling birth order was included as an additional covariate in model 2, and cluster-robust (sandwich) standard errors were used to account for within-family correlation.^22^ We conducted 3 subgroup analyses. Given that mortality risk associated with narcolepsy could vary by age and sex, we repeated models 1 and 2 stratified separately by age categories (adolescents, <18 years; adults, 18-64 years; older adults, ≥65 years) and by sex (male and female).^8,23^ Finally, to assess the influence of various psychiatric comorbidities on the association between narcolepsy and mortality, we repeated model 2 analyses by sequentially including each psychiatric comorbidity group, one group at a time. All statistical analyses were performed using SAS software, version 9.4 (SAS Institute Inc). All *P* values were from 2-tailed tests, and results were deemed statistically significant at *P* < .05.

## Results

Between 2001 and 2021, 3187 patients with narcolepsy (mean [SD] age, 29.5 [16.1] years; 1674 male patients [52.5%] and 1513 female patients [47.5%]) and 12 748 age- and sex-matched controls (mean [SD] age, 29.5 [16.1] years; 6696 male patients [52.5%] and 6052 female patients [47.5%]) were identified, with a mean [SD] follow-up of 9.4 [5.5] years (Table 1). Compared with matched controls, patients with narcolepsy exhibited a higher lifetime prevalence of psychiatric comorbidities, including neurodevelopmental disorders (544 of 3187 [17.1%] vs 315 of 12 748 [2.5%]), psychotic disorders (210 of 3187 [6.6%] vs 159 of 12 748 [1.2%]), bipolar disorders (235 of 3187 [7.4%] vs 133 of 12 748 [1.0%]), depressive disorders (1167 of 3187 [36.6%] vs 861 of 12 748 [6.8%]), anxiety disorders (1054 of 3187 [33.1%] vs 853 of 12 748 [6.7%]), obsessive-compulsive disorders (105 of 3187 [3.3%] vs 52 of 12 748 [0.4%]), eating disorders (36 of 3187 [1.1%] vs 24 of 12 748 [0.2%]), substance use disorders (52 of 3187 [1.6%] vs 100 of 12 748 [0.8%]), and personality disorders (68 of 3187 [2.1%] vs 31 of 12 748 [0.2%]). In addition, according to the CCI, a greater proportion of patients with narcolepsy than controls experienced medical comorbidities (CCI score ≥1: 2602 of 3187 [81.6%] vs 8596 of 12 748 [67.4%]). In the sibling cohorts, 2134 participants with narcolepsy and 3287 siblings without narcolepsy were included, and the same pattern of excess psychiatric and medical comorbidities was observed among those with narcolepsy.

**Table 1.** Characteristics of All Included Participants From 2001 to 2021

Characteristic	No. (%) of participants in population-based cohort			
	Matched		Sibling	
	Narcolepsy (n = 3187)	Matched controls (n = 12 748)	Narcolepsy (n = 2134)	Sibling controls (n = 3287)
Demographic information				
Age, mean (SD), y	29.5 (16.1)	29.5 (16.1)	NA	NA
Sex				
Female	1513 (47.5)	6052 (47.5)	1003 (47.0)	1633 (49.7)
Male	1674 (52.5)	6696 (52.5)	1131 (53.0)	1654 (50.3)
Personal income level, quartile				
First (highest)	759 (23.8)	2867 (22.5)	471 (22.1)	761 (23.2)
Second	741 (23.3)	3136 (24.6)	532 (24.9)	825 (25.1)
Third	586 (18.4)	2570 (20.2)	413 (19.4)	619 (18.8)
Fourth (lowest)	795 (24.9)	2841 (22.3)	505 (23.7)	660 (20.1)
Unknown	306 (9.6)	1334 (10.5)	213 (10.0)	422 (12.8)
Personal urbanization level				
Level 1 (urban)	1782 (55.9)	6937 (54.4)	1196 (56.0)	1901 (57.8)
Level 2	1157 (36.3)	4655 (36.5)	769 (36.0)	1137 (34.6)
Level 3	189 (5.9)	861 (6.8)	130 (6.1)	198 (6.0)
Level 4 (rural)	35 (1.1)	125 (1.0)	24 (1.1)	25 (0.8)
Unknown	24 (0.8)	170 (1.3)	15 (0.7)	26 (0.8)
Psychiatric comorbidities				
Neurodevelopmental disorders	544 (17.1)	315 (2.5)	412 (19.3)	188 (5.7)
Psychotic disorders	210 (6.6)	159 (1.2)	135 (6.3)	33 (1.0)
Bipolar disorders	235 (7.4)	133 (1.0)	149 (7.0)	45 (1.4)
Depressive disorders	1167 (36.6)	861 (6.8)	756 (35.4)	319 (9.7)
Anxiety disorders	1054 (33.1)	853 (6.7)	713 (33.4)	319 (9.7)
Obsessive-compulsive disorders	105 (3.3)	52 (0.4)	70 (3.3)	17 (0.5)
Eating disorders	36 (1.1)	24 (0.2)	22 (1.0)	10 (0.3)
Substance use disorders	52 (1.6)	100 (0.8)	24 (1.1)	19 (0.6)
Personality disorders	68 (2.1)	31 (0.2)	50 (2.3)	19 (0.6)
Charlson Comorbidity Index (No. of comorbidities)				
0	585 (18.4)	4152 (32.6)	475 (22.3)	1002 (30.5)
1-2	1543 (48.4)	6073 (47.6)	1211 (56.7)	1823 (55.5)
>2	1059 (33.2)	2523 (19.8)	448 (21.0)	462 (14.1)

Abbreviation: NA, not applicable.

A total of 132 patients with narcolepsy and 456 matched controls died during follow-up, yielding crude mortality rates of 44.3 per 10 000 person-years among patients with narcolepsy and 38.1 per 10 000 person-years among controls (Table 2). The Figure illustrates Kaplan-Meier survival curves stratified by narcolepsy status over the study period, while eTable 2 in Supplement 1 reports yearly cumulative incidences and corresponding risk differences. Both crude (model 1) and adjusted (model 2) HRs indicated that patients with narcolepsy did not have a significantly elevated risk of all-cause mortality compared with controls without narcolepsy (model 1: HR, 1.16; 95% CI, 0.96-1.41; model 2: HR, 0.96; 95% CI, 0.79-1.17) (Table 2). Similarly, no significantly increased risk was observed for mortality from specific causes, including natural causes (model 1: HR, 1.14; 95% CI, 0.92-1.40; model 2: HR, 0.90; 95% CI, 0.73-1.11), unnatural causes (model 1: HR, 1.34; 95% CI, 0.80-2.25; model 2: HR, 1.41; 95% CI, 0.83-2.40), accidents (model 1: HR, 1.46; 95% CI, 0.65-3.28; model 2: HR, 1.37; 95% CI, 0.64-2.95), or suicides (model 1: HR, 1.29; 95% CI, 0.61-2.73; model 2: HR, 1.41; 95% CI, 0.62-3.22).

**Table 2.** Risk of All-Cause and Cause-Specific Mortality Between Patients With Narcolepsy and Controls Without Narcolepsy

Outcome	Narcolepsy		Controls		Hazard ratio (95% CI)	
	Event, No. (%)	Mortality rate per 10 000 person-years	Event, No. (%)	Mortality rate per 10 000 person-years	Crude (model 1)	Adjusted (model 2)
Primary analysis, No.^a^	3187		2748			
Death from all causes	132 (4.1)	44.3	456 (3.6)	38.1	1.16 (0.96-1.41)	0.96 (0.79-1.17)
Death from natural causes	113 (3.5)	37.9	399 (3.1)	33.4	1.14 (0.92-1.40)	0.90 (0.73-1.11)
Death from unnatural causes	19 (0.6)	6.4	57 (0.4)	4.8	1.34 (0.80-2.25)	1.41 (0.83-2.40)
Accidents	9 (0.3)	3.0	28 (0.2)	2.3	1.46 (0.65-3.28)	1.37 (0.64-2.95)
Suicides	8 (0.3)	2.7	22 (0.2)	1.8	1.29 (0.61-2.73)	1.41 (0.62-3.22)
**Sensitivity analysis 1^a^**						
Sibling cohort, No.	2134		3287			
Death from all causes	22 (1.0)	4.7	29 (0.9)	4.0	1.17 (0.68-2.01)	1.14 (0.63-2.06)
Death from natural causes	9 (0.4)	1.9	18 (0.5)	2.5	0.77 (0.35-1.72)	0.66 (0.28-1.56)
Death from unnatural causes	13 (0.6)	2.8	11 (0.3)	1.5	1.82 (0.81-4.06)	2.08 (0.87-4.98)
Accidents	6 (0.3)	1.3	6 (0.2)	0.8	1.54 (0.50-4.77)	1.61 (0.48-5.37)
Suicides	6 (0.3)	1.3	4 (0.1)	0.6	2.31 (0.65-8.18)	3.43 (0.88-13.28)
**Sensitivity analysis 2^a^**						
Excluding data after 2020, No.	2577		10 308			
Death from all causes	89 (3.5)	42.6	339 (3.3)	40.5	1.05 (0.83-1.33)	0.88 (0.69-1.11)
Death from natural causes	77 (3.0)	36.8	291 (2.8)	34.7	1.06 (0.82-1.36)	0.84 (0.65-1.08)
Death from unnatural causes	12 (0.5)	5.7	48 (0.5)	5.7	1.00 (0.53-1.89)	1.06 (0.56-2.02)
Accidents	7 (0.3)	3.3	23 (0.2)	2.7	1.22 (0.52-2.84)	1.31 (0.55-3.10)
Suicides	3 (0.1)	1.4	18 (0.2)	2.1	0.67 (0.20-2.27)	0.62 (0.18-2.12)
**Sensitivity analysis 3^a^**						
Excluding missing data, No.	2866		11 325			
Death from all causes	106 (3.7)	37.9	378 (3.3)	34.2	1.11 (0.89-1.38)	0.96 (0.77-1.20)
Death from natural causes	92 (3.2)	32.9	330 (2.9)	29.8	1.10 (0.88-1.39)	0.93 (0.73-1.17)
Death from unnatural causes	14 (0.5)	5.0	48 (0.4)	4.3	1.15 (0.64-2.09)	1.18 (0.64-2.16)
Accidents	7 (0.2)	2.5	25 (0.2)	2.3	1.11 (0.48-2.56)	1.15 (0.49-2.71)
Suicides	6 (0.2)	2.1	19 (0.2)	1.7	1.25 (0.50-3.13)	1.24 (0.49-3.16)

^a^ Primary analysis and sensitivity analysis 2 and 3 adjusted for all matching variables (birth year, sex, income level, urbanization level, and Charlson Comorbidity Index). Sensitivity analysis 1 adjusted for all matching variables (birth year, sex, income level, urbanization level, and Charlson Comorbidity Index) and sibling birth order.

**Figure.**
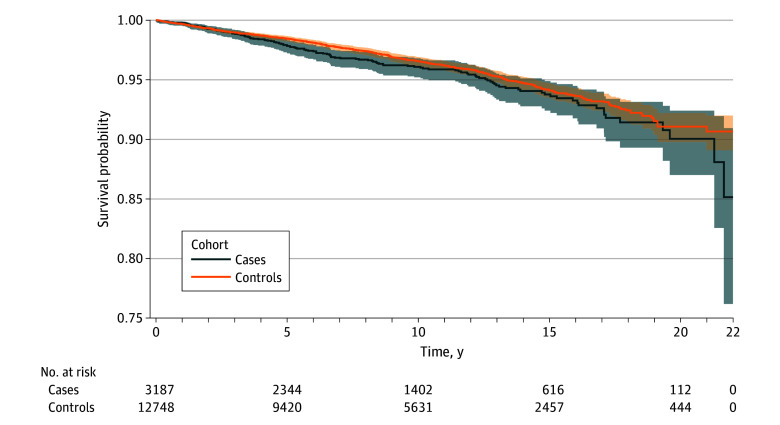
Kaplan-Meier Survival Curves for All-Cause Mortality Among Patients With Narcolepsy and Controls Without Narcolepsy Cases comprise patients with narcolepsy, and controls comprise matched controls. Shaded areas indicate 95% CIs.

In the sibling cohorts, 22 participants with narcolepsy and 29 siblings without narcolepsy died, corresponding to crude mortality rates of 4.7 per 10 000 person-years among patients with narcolepsy and 4.0 per 10 000 person-years among siblings without narcolepsy (Table 2). The analyses showed no significant differences between patients with narcolepsy and siblings without narcolepsy regarding the risks of all-cause mortality (model 1: HR, 1.17; 95% CI, 0.68-2.01; model 2: HR, 1.14; 95% CI, 0.63-2.06), death from natural causes (model 1: HR, 0.77; 95% CI, 0.35-1.72; model 2: HR, 0.66; 95% CI, 0.28-1.56), death from unnatural causes (model 1: HR, 1.82; 95% CI, 0.81-4.06; model 2: HR, 2.08; 95% CI, 0.87-4.98), death from accidents (model 1: HR, 1.54; 95% CI, 0.50-4.77; model 2: HR, 1.61; 95% CI, 0.48-5.37), or death from suicides (model 1: HR, 2.31; 95% CI, 0.65-8.18; model 2: HR, 3.43; 95% CI, 0.88-13.28). Two additional sensitivity analyses also revealed no significant differences in all-cause or cause-specific mortality between patients with narcolepsy and their matched controls.

In analyses stratified by age (Table 3), the older adult subgroup (aged ≥65 years) with narcolepsy showed higher crude risks of all-cause mortality (model 1: HR, 1.41; 95% CI, 1.09-1.83) and mortality from natural causes (model 1: HR, 1.44; 95% CI, 1.10-1.87) compared with controls without narcolepsy; however, these differences were not significant after adjustment for covariates (model 2 for all-cause mortality: HR, 1.08; 95% CI, 0.83-1.41; model 2 for natural-cause mortality: HR, 1.09; 95% CI, 0.83-1.42). No increased risk was found for other specific causes of death in this older subgroup. In addition, no significant differences in mortality risks were observed between patients with narcolepsy and controls without narcolepsy in either adolescent or adult subgroups (Table 3). Analyses stratified by sex also revealed no significant increase in the risk of all-cause or cause-specific mortality among patients with narcolepsy compared with controls without narcolepsy in either the male or female subgroups (eTable 3 in Supplement 1). Further adjustment for psychiatric comorbidities yielded results similar to the primary analyses, with no significantly elevated risk estimates for all-cause or cause-specific mortality associated with narcolepsy (Table 4).

**Table 3.** Risk of All-Cause and Cause-Specific Mortality Between Patients With Narcolepsy and Controls Without Narcolepsy, by Age

Outcome	Hazard ratio (95% CI)	
	Crude (model 1)	Adjusted (model 2)^a^
**Adolescents (<18 y)**		
Death from all causes	1.32 (0.52-3.33)	1.11 (0.43-2.82)
Death from natural causes	1.13 (0.24-5.45)	0.79 (0.16-3.94)
Death from unnatural causes	1.44 (0.46-4.53)	1.41 (0.45-4.47)
Accidents	1.32 (0.27-6.55)	1.27 (0.25-6.40)
Suicides	1.99 (0.36-10.85)	1.78 (0.32-10.02)
**Adults (aged 18-64 y)**		
Death from all causes	1.04 (0.77-1.41)	0.84 (0.62-1.14)
Death from natural causes	0.94 (0.66-1.33)	0.73 (0.51-1.04)
Death from unnatural causes	1.48 (0.80-2.73)	1.53 (0.81-2.86)
Accidents	1.50 (0.59-3.84)	1.61 (0.61-4.21)
Suicides	1.34 (0.53-3.37)	1.34 (0.52-3.45)
**Older adults (aged ≥65 y)**		
Death from all causes	1.41 (1.09-1.83)^b^	1.08 (0.83-1.41)
Death from natural causes	1.44 (1.10-1.87)^b^	1.09 (0.83-1.42)
Death from unnatural causes	0.58 (0.07-4.67)	0.69 (0.08-6.16)
Accidents	0.78 (0.09-6.52)	0.74 (0.08-6.44)
Suicides^c^	NA	NA

Abbreviation: NA, not applicable.

^a^ Adjusted for all matching variables (birth year, sex, income level, urbanization level, and Charlson Comorbidity Index).

^b^ Statistically significant.

^c^ No events occurred for either the patients with narcolepsy or the controls without narcolepsy.

**Table 4.** Risk of All-Cause and Cause-Specific Mortality Between Patients With Narcolepsy and Controls Without Narcolepsy, by Psychiatric Comorbidity Group

Outcome	Disorders, adjusted hazard ratio (95% CI)^a^								
	Neurodevelopmental	Psychotic	Bipolar	Depressive	Anxiety	Obsessive-compulsive	Eating	Substance use	Personality
Death from all causes	0.92 (0.75-1.12)	0.93 (0.76-1.14)	0.94 (0.77-1.14)	0.88 (0.72-1.08)	0.94 (0.77-1.15)	0.96 (0.79-1.17)	0.96 (0.79-1.16)	0.95 (0.78-1.16)	0.94 (0.77-1.14)
Death from natural causes	0.87 (0.70-1.08)	0.88 (0.71-1.09)	0.89 (0.72-1.10)	0.86 (0.69-1.07)	0.90 (0.72-1.11)	0.90 (0.73-1.11)	0.89 (0.72-1.10)	0.89 (0.72-1.10)	0.89 (0.72-1.10)
Death from unnatural causes	1.28 (0.73-2.22)	1.22 (0.70-2.11)	1.19 (0.68-2.07)	0.91 (0.51-1.62)	1.11 (0.63-1.95)	1.41 (0.82-2.40)	1.42 (0.84-2.42)	1.42 (0.84-2.42)	1.24 (0.72-2.16)
Accidents	1.46 (0.67-3.16)	1.30 (0.60-2.84)	1.29 (0.59-2.82)	1.27 (0.57-2.87)	1.18 (0.52-2.64)	1.41 (0.65-3.03)	1.38 (0.64-2.96)	1.38 (0.64-2.97)	1.40 (0.65-3.00)
Suicides	1.05 (0.43-2.56)	1.15 (0.48-2.71)	0.95 (0.39-2.31)	0.66 (0.27-1.61)	1.00 (0.41-2.41)	1.34 (0.58-3.11)	1.42 (0.62-3.24)	1.42 (0.62-3.24)	1.10 (0.46-2.64)

^a^ Adjusted for all matching variables (birth year, sex, income level, urbanization level, and Charlson Comorbidity Index).

## Discussion

This study investigated a total of 3187 patients with a diagnosis of narcolepsy and 12 748 matched controls without narcolepsy in Taiwan, with a follow-up period spanning 22 years. The adjusted HR for all-cause mortality was 0.96 (95% CI, 0.79-1.17), and HR estimates were 0.90 (95% CI, 0.73-1.11) for natural deaths, 1.41 (95% CI, 0.83-2.40) for unnatural deaths, 1.37 (95% CI, 0.64-2.95) for accidental deaths, and 1.41 (95% CI, 0.62-3.22) for suicides; all HRs had wide 95% CIs that spanned both decreases and increases in risk, providing no clear evidence of excess mortality. These observations remained consistent in the sibling cohort analysis and 2 additional sensitivity analyses. Stratified analyses by sex and psychiatric comorbidities similarly demonstrated no significant association of these factors with mortality risk among patients with narcolepsy. However, subgroup analysis by age indicated a higher crude mortality rate among older adults, although this increase was attenuated after adjustment for birth year, sex, income level, urbanization, and CCI. In addition, mortality risks in adolescent and adult subgroups were not significantly higher.

Evidence on mortality and cause of death in narcolepsy is limited. A US claims study observed a significantly higher annual mortality rate (approximately 1.5-fold) among patients with narcolepsy compared with the general population over a 3-year period.^8^ This discrepancy with our findings could be attributed to several factors. First, previous studies did not match patients with narcolepsy with controls by age and sex, resulting in dissimilar comparison groups. Second, differences in mortality risk may exist due to ethnic or regional variations (North America vs Asia). In contrast, a Danish study with methods closely resembling our methods compared patients with narcolepsy with matched healthy controls over a 12-year period, reporting slightly but not significantly increased crude mortality risk (HR, 1.25).^9^ Our study further controlled for potential confounders using the CCI and used sibling cohorts as comparison groups, reinforcing the robustness of our findings that narcolepsy was not materially associated with increased all-cause or cause-specific mortality.

Our analysis also assessed mortality from unnatural causes—accidents and suicides—and again detected no significant association with excess deaths from unnatural causes among patients with narcolepsy. Prior reports noted more accidental injuries among patients with narcolepsy,^24^ fueling worries of higher accidental deaths, yet others argued that patients adopt safety behaviors after diagnosis or initial mishaps,^24^ blunting risk. The results of our study support this hypothesis, implying that individuals with narcolepsy may, for example, limit driving, learn to stop activities at the first sign of sleepiness, or receive adequate treatment that mitigates accident-related harm. Regarding suicide, while previous studies and our own indicated higher comorbidity with depression and possibly increased suicidal ideation among patients with narcolepsy,^12,25,26^ we observed no significantly elevated risk of suicide mortality associated with narcolepsy. Stratified analysis of patients with psychiatric comorbidities, particularly depression, further supported this conclusion (Table 4). It is plausible that patients with narcolepsy with comorbidities of depressive disorder in clinical settings may receive adequate medical interventions, thereby neutralizing potential increases in risk of suicide death.^12^ Nevertheless, given our study’s limited number of death events and potential lack of statistical power, further subgroup analyses were not feasible. Future studies with larger samples are recommended for deeper exploration of these issues.

When restricting analyses to older adults, we noted that only participants aged 65 years or older showed a significantly higher crude HR for all-cause mortality. This finding echoes a 17-year Danish cohort that found excess mortality among older patients (≥60 years) with narcolepsy (HR, 1.38; 95% CI, 1.12-1.69) but not among those aged 20 to 59 years.^23^ In our data, excess deaths were associated with natural causes (Table 3) and decreased to an HR of 1.09 (95% CI, 0.83-1.42) after multivariable adjustment, including for medical comorbidities. These patterns imply that older individuals with narcolepsy have heavier burdens of conditions—particularly cardiovascular disease—that elevate rates of death from natural caused; narcolepsy itself seems unlikely to be associated with additional risk once comorbidity is considered. Mortality estimates stratified by individual psychiatric disorders paralleled the primary analysis, indicating that psychiatric comorbidities did not materially modify the mortality gap between individuals with narcolepsy and matched comparators.

### Limitations

Several limitations should be considered. First, key lifestyle and occupational factors associated with mortality—such as diet, smoking, and work-related exposures—were not captured in the registry, precluding adjustment for these potential confounders. Second, cause-specific deaths were relatively infrequent, particularly in the sibling cohort and in other sensitivity analyses, reducing statistical power for these outcomes. Third, the database does not reliably distinguish narcolepsy with cataplexy from narcolepsy without cataplexy; the latter may represent an early stage of the former,^27^ complicating precise phenotypic classification and limiting subgroup analyses. Fourth, although polysomnography and multiple sleep-latency testing are routinely used in Taiwan to confirm narcolepsy, objective sleep examination results are not recorded in the NHIRD. To enhance diagnostic certainty, cases were therefore defined as individuals receiving 2 or more outpatient or inpatient narcolepsy diagnoses from board-certified neurologists or psychiatrists. Fifth, the cohort was relatively young at baseline (mean [SD] age, 29.5 [16.1] years), so even after 22 years of follow-up, substantial mortality would be unexpected; a longer observation will be required to detect late deaths associated with comorbid conditions such as hypertension or obesity, which are common in narcolepsy. Sixth, because the study was conducted exclusively in Taiwan, the findings may not be generalizable to populations with different health care systems, clinical practices, or lifestyle patterns.

## Conclusions

In this cohort study of Taiwanese residents, narcolepsy was not associated with excess all-cause or cause-specific (natural, unnatural, accidental, and suicide causes) mortality vs matched controls or siblings without narcolepsy. Crude rates were higher among older adults but attenuated after adjusting for comorbidities, and estimates remained imprecise. Sex and psychiatric comorbidity were not associated with an alteration in risk. Overall, findings are reassuring yet cannot exclude a modest increase in mortality; continued monitoring and replication in other populations with longer follow-up are advisable.

## Notes

Supplement 1. **eTable 1.** The Diagnostic Codes of Medical Comorbidities **eTable 2.** Cumulative Incidence and Risk Differences of All-Cause Mortality **eTable 3.** The Risk of All-Cause and Cause-Specific Mortality Between Narcolepsy Patients and Unaffected Controls, by Sex **eFigure.** Flowchart of the Selection Process for the Cohort Study

Supplement 2.
Data Sharing Statement
