# Supplementary material for: All-Cause and Cause-Specific Mortality Among Patients With Narcolepsy
Source: JAMA Netw Open. 2025 Oct 9;8(10):e2536771. doi: 10.1001/jamanetworkopen.2025.36771 (PMC12511998; doi:10.1001/jamanetworkopen.2025.36771)
Supplement: Supplement 1. — eTable 1. The Diagnostic Codes of Medical Comorbidities eTable 2. Cumulative Incidence and Risk Differences of All-Cause Mortality eTable 3. The Risk of All-Cause and Cause-Specific Mortality Between Narcolepsy Patients and Unaffected Controls, by Sex eFigure. Flowchart of the Selection Process for the Cohort Study [file jamanetwopen-e2536771-s001.pdf]

Supplementary Online Content

Hsu CW, Yang YS, Chen YCB, et al. All-cause and cause-specific mortality among patients with narcolepsy. *JAMA Netw Open.* 2025;8(10):e2536771. doi:10.1001/jamanetworkopen.2025.36771

eTable 1. The Diagnostic Codes of Medical Comorbidities
eTable 2. Cumulative Incidence and Risk Differences of All-Cause Mortality
eTable 3. The Risk of All-Cause and Cause-Specific Mortality Between Narcolepsy Patients and Unaffected Controls, by Sex
eFigure. Flowchart of the Selection Process for the Cohort Study

This supplementary material has been provided by the authors to give readers additional information about their work.

© 2025 Hsu CW et al. *JAMA Network Open.*

eTable 1. The Diagnostic Codes of Medical Comorbidities

Medical comorbidities	International Classification of Diseases 9th and 10th
Neurodevelopmental disorders	
(intellectual disability)	317-319; F70–F73, F78, F79
(autism spectrum disorders)	299; F84
(attention-deficit/hyperactivity disorder)	314; F90
(Tourette syndrome and tic disorder)	307.2; F95
Psychotic disorders	295, 297, 298.1, 298.3, 298.4, 298.8, 298.9; F20, F22-F25, F28, F29
Bipolar disorders	296.0, 296.1, 296.4–296.7, 296.80, 296.81, 296.89, 301.13; F30, F31, F34.0
Depressive disorders	296.2, 296.3, 300.4, 311, 625.4; F32, F33, F34.1
Anxiety disorders	300.0, 300.2, 309.21; F40, F41, F93.0
Obsessive-compulsive disorders	300.3; F42
Eating disorders	307.1, 307.5; F50
Substance use disorders	303, 304, 305.0, 305.2, 305.3, 305.4, 305.5, 305.6, 305.7, 305.8, 305.9; F10-F16, F18, F19
Personality disorders	301; F60

© 2025 Hsu CW et al. *JAMA Network Open.*

eTable 2. Cumulative Incidence and Risk Differences of All-Cause Mortality

Year	Narcolepsy (n = 3187)	Matched control (n = 12,748)	Risk differences (case - control)
**0**	100.0 (100.0–100.0)	100.0 (100.0–100.0)	0.0 (0.0–0.0)
**1**	99.8 (99.5–99.9)	99.7 (99.6–99.8)	0.1 (-0.1–0.3)
**2**	99.3 (98.9–99.5)	99.3 (99.1–99.4)	0.0 (-0.4–0.3)
**3**	98.9 (98.4–99.2)	99.0 (98.8–99.1)	-0.1 (-0.5–0.3)
**4**	98.4 (97.9–98.8)	98.7 (98.5–98.9)	-0.3 (-0.8–0.2)
**5**	97.9 (97.3–98.3)	98.4 (98.2–98.7)	-0.6 (-1.2–0.0)
**6**	97.4 (96.7–97.9)	98.1 (97.8–98.3)	-0.7 (-1.4–-0.1)
**7**	96.8 (96.1–97.5)	97.7 (97.4–98.0)	-0.9 (-1.6–-0.1)
**8**	96.7 (95.9–97.3)	97.4 (97.1–97.7)	-0.7 (-1.5–0.1)
**9**	96.2 (95.4–96.9)	96.9 (96.5–97.2)	-0.7 (-1.5–0.2)
**10**	96.1 (95.2–96.8)	96.6 (96.2–96.9)	-0.5 (-1.4–0.4)
**11**	95.9 (95.0–96.6)	96.2 (95.8–96.6)	-0.3 (-1.2–0.6)
**12**	95.4 (94.4–96.3)	95.9 (95.4–96.3)	-0.4 (-1.4–0.6)
**13**	94.5 (93.3–95.5)	95.3 (94.7–95.8)	-0.8 (-1.9–0.4)
**14**	94.1 (92.8–95.1)	94.7 (94.1–95.3)	-0.7 (-2.0–0.6)
**15**	93.8 (92.4–94.9)	94.1 (93.4–94.7)	-0.3 (-1.7–1.0)
**16**	93.3 (91.8–94.5)	93.7 (93.0–94.3)	-0.4 (-1.9–1.1)
**17**	92.6 (90.9–94.0)	93.2 (92.4–93.9)	-0.6 (-2.3–1.1)
**18**	91.4 (89.3–93.1)	92.4 (91.5–93.3)	-1.0 (-3.1–1.1)
**19**	91.4 (89.3–93.1)	91.5 (90.3–92.6)	-0.1 (-2.3–2.1)
**20**	90.0 (87.0–92.4)	91.1 (89.8–92.2)	-1.0 (-4.0–1.9)
**21**	90.0 (87.0–92.4)	90.7 (89.1–92.0)	-0.6 (-3.7–2.5)

Data was expressed as cumulative incidence rate, percentage (95% confidence interval).

© 2025 Hsu CW et al. *JAMA Network Open.*

eTable 3. The Risk of All-Cause and Cause-Specific Mortality Between Narcolepsy Patients and Unaffected Controls, by Sex

Characteristics	Crude hazard ratio	Adjusted hazard ratio
**Male**		
Death from all causes	1.10 (0.87–1.40)	0.92 (0.72–1.17)
Death from natural causes	1.06 (0.82–1.37)	0.83 (0.64–1.08)
Death from unnatural causes	1.41 (0.76–2.58)	1.49 (0.80–2.78)
Accidents	1.53 (0.68–3.46)	1.63 (0.71–3.73)
Suicides	1.15 (0.38–3.48)	1.09 (0.35–3.38)
**Female**		
Death from all causes	1.29 (0.92–1.80)	1.12 (0.80–1.56)
Death from natural causes	1.30 (0.92–1.85)	1.09 (0.77–1.56)
Death from unnatural causes	1.18 (0.43–3.19)	1.29 (0.47–3.55)
Accidents	0.57 (0.07–4.66)	0.62 (0.07–5.24)
Suicides	2.00 (0.60–6.65)	1.96 (0.58–6.64)

[1] Adjusted for all matching variables (birth year, sex, income level, urbanization level, and Charlson Comorbidity Index).

© 2025 Hsu CW et al. *JAMA Network Open.*

eFigure. Flowchart of the Selection Process for the Cohort Study

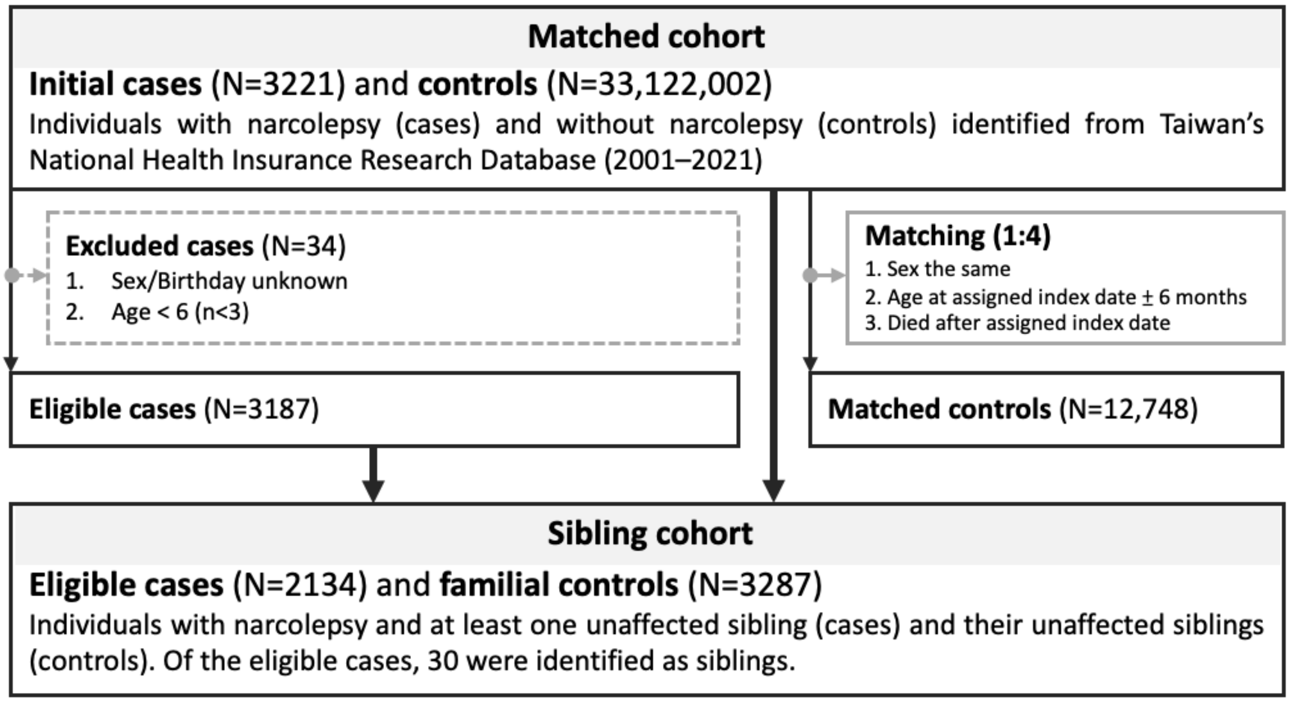

© 2025 Hsu CW et al. *JAMA Network Open.*
