# Supplementary material for: All-Cause and Cause-Specific Mortality Among Patients With Narcolepsy
Source: JAMA Netw Open. 2025 Oct 9;8(10):e2536771. doi: 10.1001/jamanetworkopen.2025.36771 (PMC12511998; doi:10.1001/jamanetworkopen.2025.36771)
Supplement: Supplement 2. — Data Sharing Statement [file jamanetwopen-e2536771-s002.pdf]

Data Sharing Statement

Hsu. All-Cause and Cause-Specific Mortality Among Patients With Narcolepsy. *JAMA Netw Open*. Published October 09, 2025. doi:10.1001/jamanetworkopen.2025.36771

Data
Data available: No

Additional Information
Explanation for why data not available: The data utilized in this study were derived from the Taiwan National Health Insurance Research Database. Access to these data requires approval from the Institutional Review Board and permission from the Taiwan Ministry of Health and Welfare; therefore, the data cannot be made publicly available by the authors.
